# Supplementary figures and images for: Functionally distinct BMP1 isoforms show an opposite pattern of abundance in plasma from non-small cell lung cancer subjects and controls
Source: PLoS One. 2023 Mar 29;18(3):e0282821. doi: 10.1371/journal.pone.0282821 (PMC10058078; doi:10.1371/journal.pone.0282821)

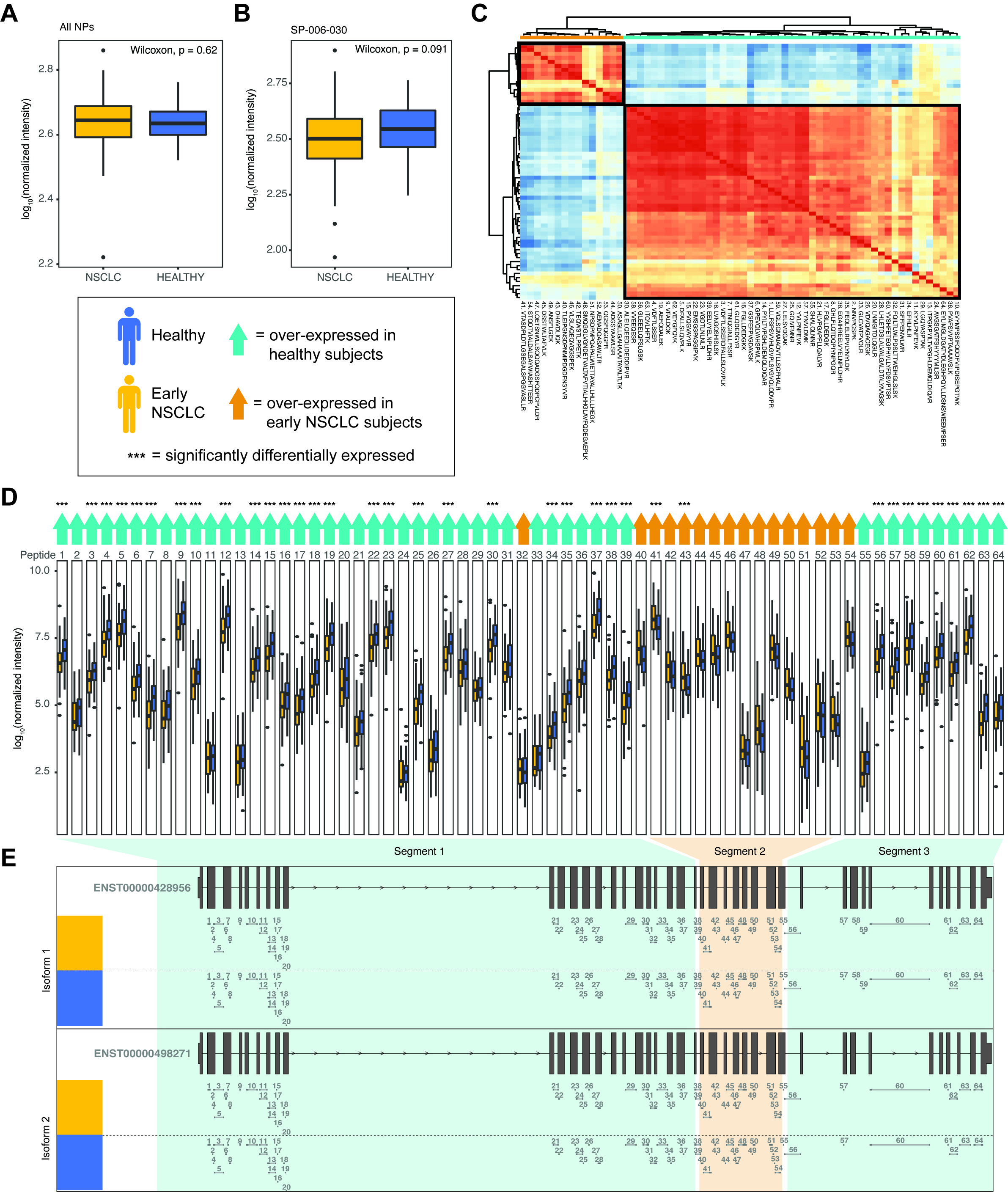

Supplement: S1 Fig — A. Box plot showing the log10 median normalized intensities of C4A in early NSCLC subjects (yellow) and in healthy subjects (blue) with collapsed abundances across NPs. P-values, calculated using a Wilcoxon test, are shown. B. Box plot showing the log10 median normalized intensities of C4A in early NSCLC subjects (yellow) and in healthy subjects (blue) in NP, SP-006-030. P-values, calculated using a Wilcoxon test, are shown. C. Heatmap showing the Pearson correlation of the 64 C4A peptide abundances, where low correlation is indicated in shades of blue and high correlation is indicated in shades of red. Correlation values were clustered using hierarchical clustering. Peptides are annotated by the direction of DE, including over-expressed in healthy subjects are highlighted in teal and early NSCLC are highlighted in orange. D. Series of boxplots showing the log10 median normalized intensities of 64 peptides mapping C4A in early NSCLC (yellow) and healthy subjects (blue). Peptides that are over-expressed in healthy subjects are indicated with a teal arrow and in early NSCLC are indicated with an orange arrow. Peptides that are significantly DE are indicated with a triple asterisk. P-values, calculated using a Wilcoxon test and adjusted, are shown. E. Gene structure plots of 2 known C4A protein coding transcripts (i.e., isoforms) with the 64 C4A peptides mapped to genomic region. Peptides spanning intronic regions are indicated with a horizontal line. Peptides 1–39 (except peptide 32), corresponding to being over-expressed in healthy subjects, are boxed in teal, creating one segment. Peptides 40–53, corresponding to being over-expressed early NSCLC, are boxed in orange, creating a second segment. Peptides 54–63 (except peptide 64), corresponding to being over-expressed in healthy subjects, are boxed in teal, creating a third segment. Segment patterns do not appear to correspond to any known protein isoforms, potentially indicating a novel isoform. (TIF) [file pone.0282821.s001.tif]

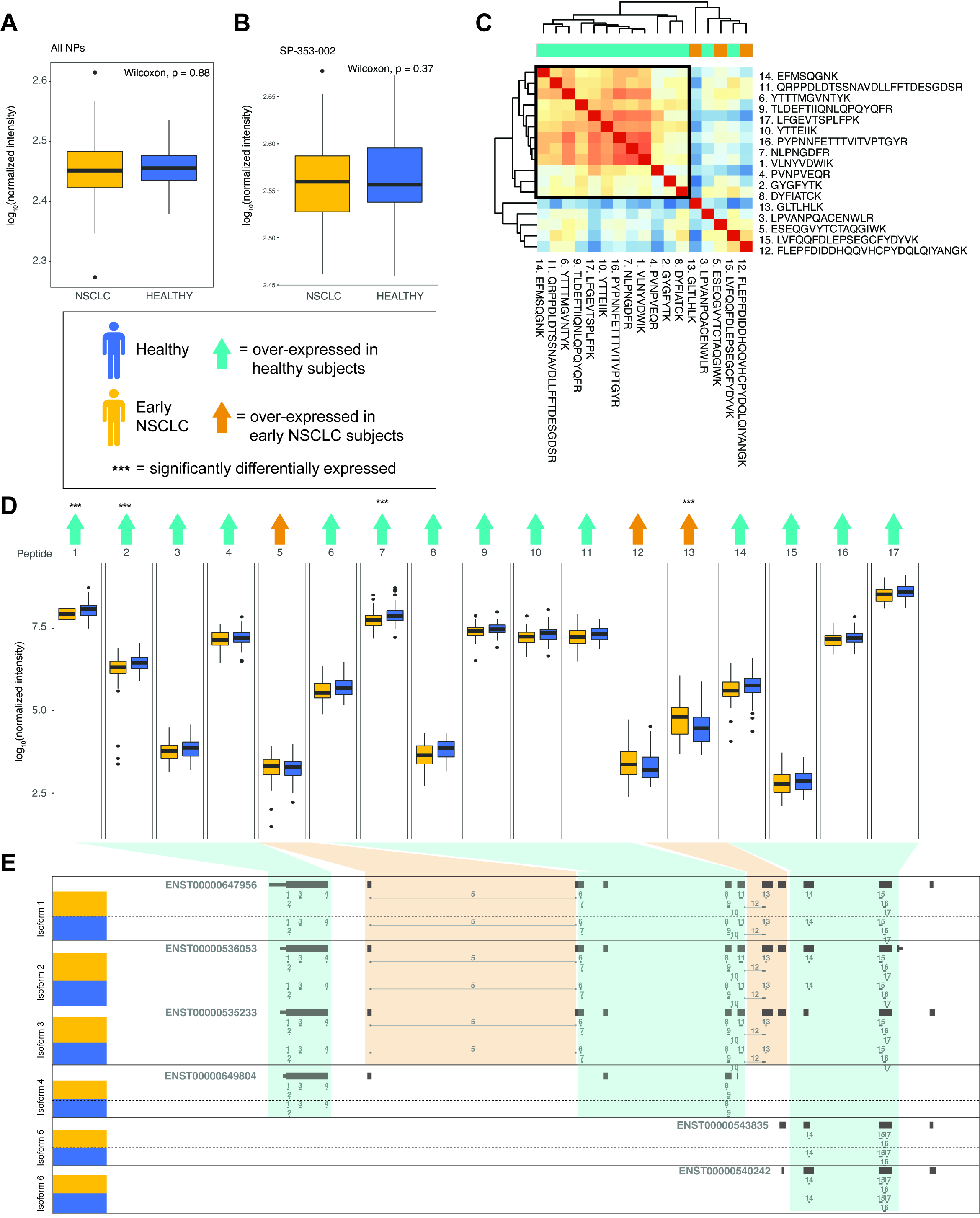

Supplement: S2 Fig — A. Box plot showing the log10 median normalized intensities of C1R in early NSCLC subjects (yellow) and in healthy subjects (blue) with collapsed abundances across NPs. P-values, calculated using a Wilcoxon test, are shown. B. Box plot showing the log10 median normalized intensities of C1R in early NSCLC subjects (yellow) and in healthy subjects (blue) in NP, SP-353-002. P-values, calculated using a Wilcoxon test, are shown. C. Heatmap showing the Pearson correlation of the 17 C1R peptide abundances, where low correlation is indicated in shades of blue and high correlation is indicated in shades of red. Correlation values were clustered using hierarchical clustering. Peptides are annotated by the direction of DE, including over-expressed in healthy subjects are highlighted in teal and early NSCLC are highlighted in orange. D. Series of boxplots showing the log10 median normalized intensities of 17 peptides mapping C1R in early NSCLC (yellow) and healthy subjects (blue). Peptides that are over-expressed in healthy subjects are indicated with a teal arrow and in early NSCLC are indicated with an orange arrow. Peptides that are significantly DE are indicated with a triple asterisk. P-values, calculated using a Wilcoxon test and adjusted, are shown. Gene structure plots of 6 known C1R protein coding transcripts (i.e., isoforms) with the 17 C1R peptides mapped to genomic region. Peptides spanning intronic regions are indicated with a horizontal line. Peptides corresponding to being over-expressed in healthy subjects are boxed in teal. Peptides corresponding to being over-expressed early NSCLC are boxed in orange. (TIF) [file pone.0282821.s002.tif]

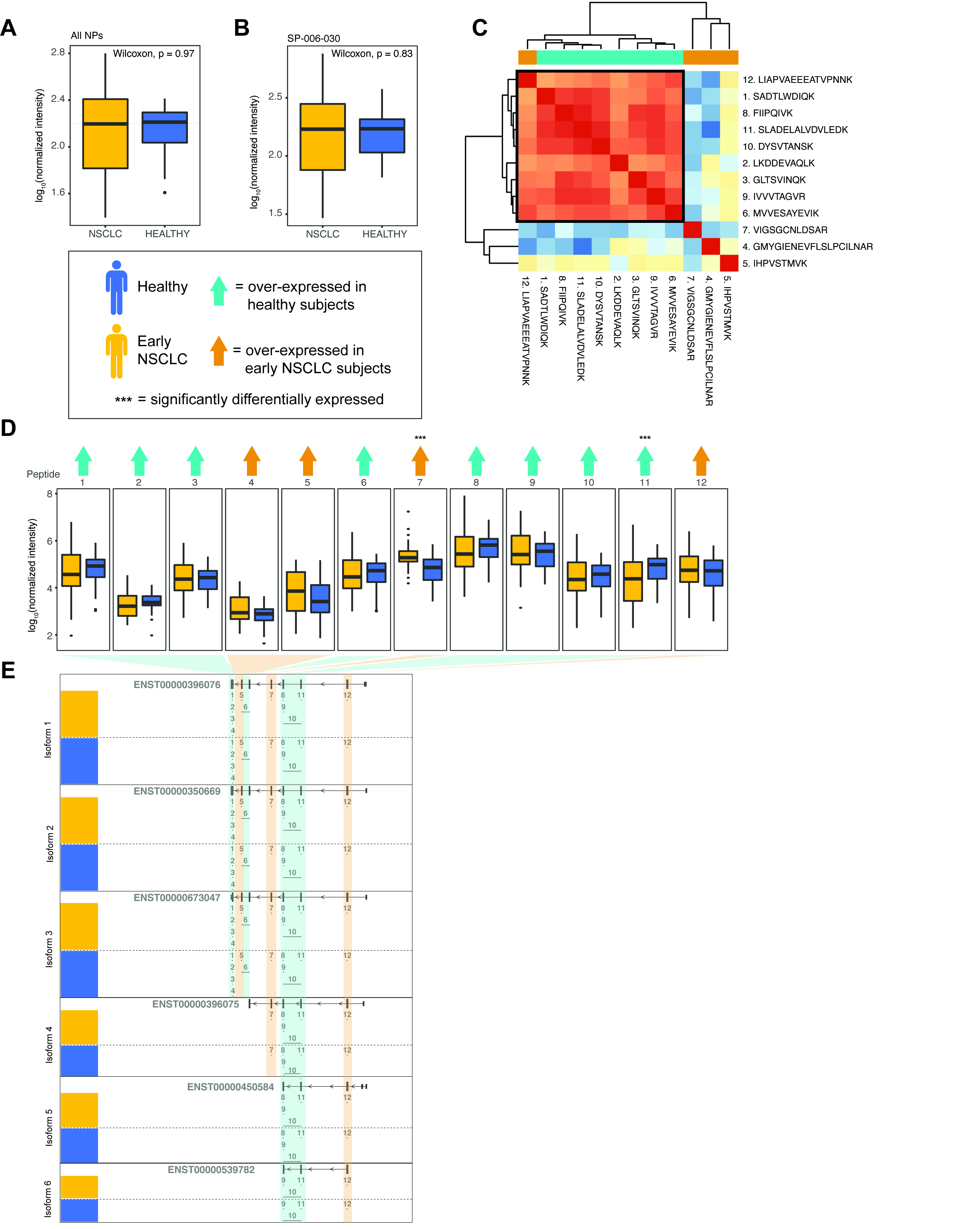

Supplement: S3 Fig — A. Box plot showing the log10 median normalized intensities of LDHB in early NSCLC subjects (yellow) and in healthy subjects (blue) with collapsed abundances across NPs. P-values, calculated using a Wilcoxon test, are shown. B. Box plot showing the log10 median normalized intensities of LDHB in early NSCLC subjects (yellow) and in healthy subjects (blue) in NP, SP-006-030. P-values, calculated using a Wilcoxon test, are shown. C. Heatmap showing the Pearson correlation of the 12 LDHB peptide abundances, where low correlation is indicated in shades of blue and high correlation is indicated in shades of red. Correlation values were clustered using hierarchical clustering. Peptides are annotated by the direction of DE, including over-expressed in healthy subjects are highlighted in teal and early NSCLC are highlighted in orange. D. Series of boxplots showing the log10 median normalized intensities of 12 peptides mapping LDHB in early NSCLC (yellow) and healthy subjects (blue). Peptides that are over-expressed in healthy subjects are indicated with a teal arrow and in early NSCLC are indicated with an orange arrow. Peptides that are significantly DE are indicated with a triple asterisk. P-values, calculated using a Wilcoxon test and adjusted, are shown. E. Gene structure plots of 6 known LDHB protein coding transcripts (i.e., isoforms) with the 12 LDHB peptides mapped to genomic region. Peptides spanning intronic regions are indicated with a horizontal line. Peptides corresponding to being over-expressed in healthy subjects are boxed in teal. Peptides corresponding to being over-expressed early NSCLC are boxed in orange. (TIF) [file pone.0282821.s003.tif]

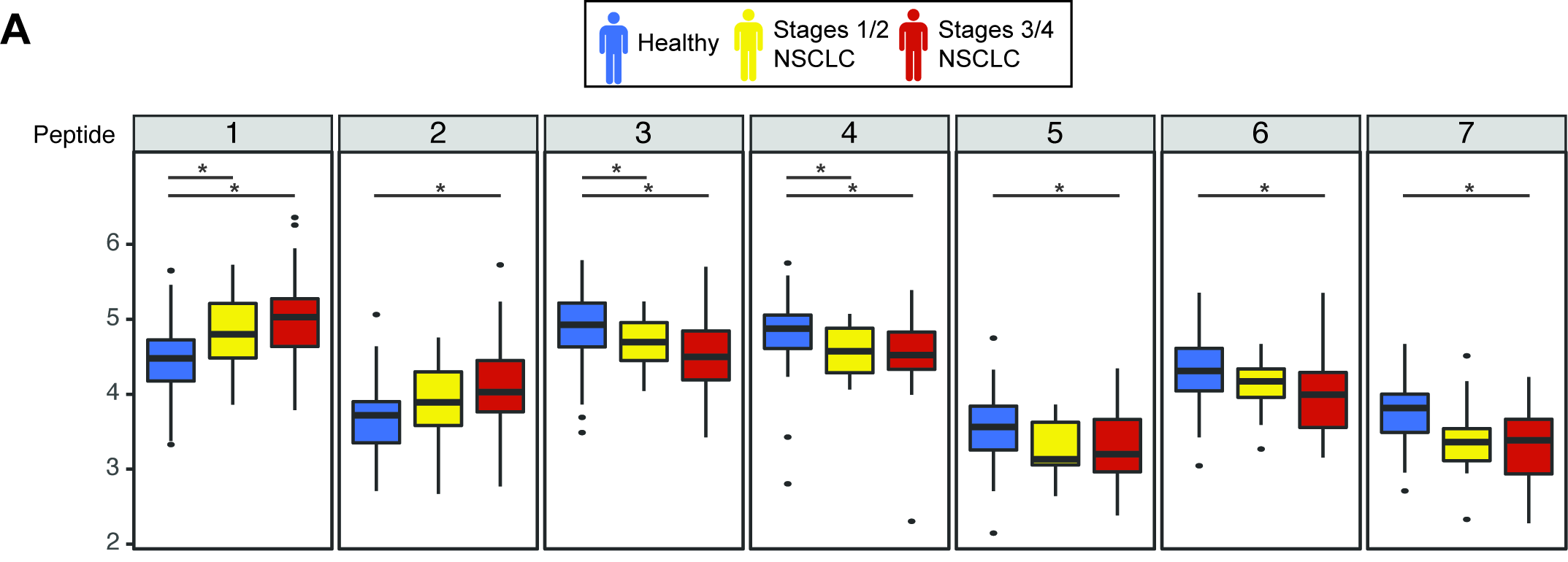

Supplement: S4 Fig — Series of boxplots showing the log10 median normalized intensities of 7 peptides mapping BMP1 in healthy subjects (blue, stages 1 and 2 NSCLC (yellow), and stages 3 and 4 (red). *Peptides that are significantly DE (Wilcoxon test and adjusted). (TIF) [file pone.0282821.s004.tif]
