# Supplementary material for: Functionally distinct BMP1 isoforms show an opposite pattern of abundance in plasma from non-small cell lung cancer subjects and controls
Source: PLoS One. 2023 Mar 29;18(3):e0282821. doi: 10.1371/journal.pone.0282821 (PMC10058078; doi:10.1371/journal.pone.0282821)
Supplement: S1 File — File containing the supplementary results and supplementary methods, with the associated supplementary references. (PDF) [file pone.0282821.s005.pdf]

Supplementary Results

High-resolution proteomics identifies known and novel biomarkers

To test the utility of NP and peptide-level interrogation of complex biological samples, we examined the top 10 most DA proteins and peptides. Among the top DA peptides, we observed peptides mapping to ITIH2, ANTXR2, and ANTXR1, which are known to be downregulated in early NSCLC plasma samples. Downregulation of ITIH2 expression has been seen in 70% of breast cancers, 71% of lung cancers, and 70% of renal tumors [1]. ANTXR2/CMG2 was shown to inhibit breast cancer cell growth and is inversely correlated with disease progression and prognosis [2]. ANTXR1 can reduce tumor growth in vivo by targeting cancer stem cells in conjunction with LeTx [3]. In agreement with results of other studies, our analysis of NSCLC plasma samples showed upregulation of well-defined pro-inflammatory and cancer biomarkers such as CRP, S100A9, and S100A8 [4,5]. Together, the observation of known hallmark cancer and inflammatory biomarkers indicates Proteograph-derived proteomic data captures known biological differences and may suggest the presence of other novel biomarkers.

In-depth examination of peptides from C4A, C1R, and LDHB isoforms

To interrogate the potential different isoforms, we next examined C4A. At the collapsed protein (Fig S1A) and NP:protein (Fig S1B) level, as for BMP1, the difference in C4A abundance is not statistically significant, however at the peptide level there are many significantly differentially expressed peptides (Fig S1D). Like BMP1, this further supports protein abundance comparisons mask differences that occur at the peptide level. Pairwise Pearson correlation and hierarchical clustering analysis showed two distinct clusters driven by peptide abundance correlation in peptides 40-54 (cluster 1) and peptides 1-39 and 55-64 (cluster 2) (Fig S1C). Mapping the peptides to the two known protein coding isoform transcripts (ENST00000428956 and ENST00000498271) and ordering them according to exon order (Fig S1E), we observed three distinct segments of corresponding direction of C4A peptide differential expression abundance. Specifically, peptides 1-39 were upregulated in healthy subjects (segment 1), peptides 40-54 were upregulated in early NSCLC subjects (segment 2), and peptides 55-64 were upregulated in healthy subjects (segment

3) (Fig S1D). Interestingly, segments 1 and 3 correspond to cluster 2 and segment 2 corresponds to cluster 1, indicating discordant expression of NSCLC-associated exons in segment 2. In contrast to results from analysis BMP1, there was no obvious association with the two known C4A protein coding isoform transcripts. However, the discordant peptides of segment 2 suggest the presence of an unknown isoform or a smaller byproduct from C4A or other members of the C4 complex. [6]

To interrogate the other potential isoforms, we next examined C1R and LDHB. At the collapsed protein (Fig S2A and S3A) and NP:protein (Fig S2B and S3B) level, the differences in C1R and LDHB abundances, respectively, were not statistically significant. However, at the peptide level we observe differentially expressed peptides (Fig S2D and S3D), indicating protein abundance comparisons mask differences that occur at the peptide level. Pairwise Pearson correlation and hierarchical clustering analysis showed one distinct cluster in each protein. For C1R, this consisted of moderately correlated peptides 1, 2, 4, 6-11, 14, 16, and 17 (cluster 1) and a weak correlation between peptides 3, 5, 12, 13, and 15 (Fig S2C). For LDHB, this consisted of highly correlated peptides 1-3, 6, and 8-11 (cluster 1) and a weak correlation between peptides 4, 5, and 7 (Fig S3C).

We mapped the peptides to the known protein coding isoform transcripts (C1R: ENST00000647956, ENST00000536053, ENST00000535233, ENST00000649804, ENST00000543835, and ENST00000540242; and LDHB: ENST00000647956, ENST00000536053, ENST00000535233, ENST00000649804, ENST00000543835, and ENST00000540242) and ordered them according to exon order (Fig S2E and S3E). There was no clear pattern in healthy or NSCLC subject peptide upregulation corresponding to any of the known isoforms for either protein. However, we did observe upregulation in C1R peptides 14-17 in healthy subjects corresponding to the two short isoforms (isoforms 5 and 6; Fig S2E). Beyond this observation, we could not explain the discordance in peptide abundances. Further examination of C1R and LDHB protein isoforms is needed to further explain the discordance in peptide abundances. Together, these results indicate discordant peptide abundance can be utilized to identify some disease-relevant protein isoforms, as was observed in the case of BMP1 and C4A. However, this approach cannot explain all discordance, as was observed in the case of C1R and LDHB. Further work may expand our understanding of disease-associated proteoforms.

Supplementary Methods

Sample collection and data generation as previously described[7]. Subjects diagnosed with NSCLC stage 1, 2, and 3 were labeled as early NSCLC. Subjects with NSCLC stage 4 were labeled as Late NSCLC. In addition, we have healthy and pulmonary comorbid control arms. Subjects diagnosed with NSCLC but with Unknown stage were removed from analysis; subjects who did not have peptides detected in all nanoparticles in the 10-NP panels were also removed. Summary statistics of protein counts and peptide counts per protein were calculated at this point.

Next, proteins were filtered to those present in at least 50% of subjects from either heathy or early cases, leaving us with a total of 188 subjects (80 control and 108 NSCLC). Peptide intensities were median normalized and natural logged.

Supplementary References

1. Hamm, A. *et al.* Frequent expression loss of Inter-alpha-trypsin inhibitor heavy chain (ITIH) genes in multiple human solid tumors: A systematic expression analysis. *BMC Cancer* **8**, 1–15 (2008).

2. Ye, L., Sun, P.-H., Malik, M. F. A., Mason, M. D. & Jiang, W. G. Capillary morphogenesis gene 2 inhibits growth of breast cancer cells and is inversely correlated with the disease progression and prognosis. *J. Cancer Res. Clin. Oncol.* **140**, 957–967 (2014).

3. Rouleau, C. *et al.* The systemic administration of lethal toxin achieves a growth delay of human melanoma and neuroblastoma xenografts: Assessment of receptor contribution. *Int. J. Oncol.* **32**, 739–748 (2008).

4. Gebhardt, C., Németh, J., Angel, P. & Hess, J. S100A8 and S100A9 in inflammation and cancer. *Biochem. Pharmacol.* **72**, 1622–1631 (2006).

5. Watson, J., Salisbury, C., Banks, J., Whiting, P. & Hamilton, W. Predictive value of inflammatory markers for cancer diagnosis in primary care: a prospective cohort study using electronic health records. *Br. J. Cancer* **120**, 1045–1051 (2019).

6. Li, N. *et al.* Association between C4, C4A, and C4B copy number variations and susceptibility to autoimmune diseases: A meta-analysis. *Sci. Rep.* **7**, 1–9 (2017).

7. Blume, J. E. *et al.* Rapid, deep and precise profiling of the plasma proteome with multi-nanoparticle protein corona. *Nat. Commun.* **11**, 1–14 (2020).
